# Navigation of guidewires and catheters in the body during intervention procedures: a review of computer-based models

**DOI:** 10.1117/1.JMI.5.1.010902

**Published:** 2018-01-29

**Authors:** Hoda Sharei, Tanja Alderliesten, John J. van den Dobbelsteen, Jenny Dankelman

**Affiliations:** a Delft University of Technology, Department of Biomechanical Engineering, Faculty of Mechanical, Maritime and Materials Engineering, Delft, The Netherlands; b Academic Medical Center, Department of Radiation Oncology, Amsterdam, The Netherlands

**Keywords:** guidewire, catheter, modeling, simulation, training, virtual reality, vascular phantom

## Abstract

Guidewires and catheters are used during minimally invasive interventional procedures to traverse in vascular system and access the desired position. Computer models are increasingly being used to predict the behavior of these instruments. This information can be used to choose the right instrument for each case and increase the success rate of the procedure. Moreover, a designer can test the performance of instruments before the manufacturing phase. A precise model of the instrument is also useful for a training simulator. Therefore, to identify the strengths and weaknesses of different approaches used to model guidewires and catheters, a literature review of the existing techniques has been performed. The literature search was carried out in Google Scholar and Web of Science and limited to English for the period 1960 to 2017. For a computer model to be used in practice, it should be sufficiently realistic and, for some applications, real time. Therefore, we compared different modeling techniques with regard to these requirements, and the purposes of these models are reviewed. Important factors that influence the interaction between the instruments and the vascular wall are discussed. Finally, different ways used to evaluate and validate the models are described. We classified the developed models based on their formulation into finite-element method (FEM), mass-spring model (MSM), and rigid multibody links. Despite its numerical stability, FEM requires a very high computational effort. On the other hand, MSM is faster but there is a risk of numerical instability. The rigid multibody links method has a simple structure and is easy to implement. However, as the length of the instrument is increased, the model becomes slower. For the level of realism of the simulation, friction and collision were incorporated as the most influential forces applied to the instrument during the propagation within a vascular system. To evaluate the accuracy, most of the studies compared the simulation results with the outcome of physical experiments on a variety of phantom models, and only a limited number of studies have done face validity. Although a subset of the validated models is considered to be sufficiently accurate for the specific task for which they were developed and, therefore, are already being used in practice, these models are still under an ongoing development for improvement. Realism and computation time are two important requirements in catheter and guidewire modeling; however, the reviewed studies made a trade-off depending on the purpose of their model. Moreover, due to the complexity of the interaction with the vascular system, some assumptions have been made regarding the properties of both instruments and vascular system. Some validation studies have been reported but without a consistent experimental methodology.

## 1 Introduction

Endovascular interventions include a variety of techniques that give access to the vascular system through small incisions. The access is mainly via guidewires and catheters. Despite the advantages of these procedures, such as decreased surgical trauma and accelerated recovery,^1^^–^^3^ new challenges are imposed on specialists. For example, they lose the direct access and the visual feedback and instead they have to manipulate the instrument (i.e., the guidewire and the catheter) from outside the body by applying a translation and/or rotation motion at its proximal side.

Traditionally, the way to learn these skills is by iterative learning on a patient. However, this incorporates a high risk for the patient and is also time-consuming. Another way is using cadavers or live animals. These methods are expensive and neither of them completely resembles an actual human vascular system. Employing phantoms is another emerging way to practice the new skills; however, the trainee is restricted to limited possible geometries. An additional drawback of the mentioned training methods is the exposure to x-ray during the training since the visual feedback is provided by x-ray imaging. Consequently, there is no single method that satisfies all the requirements.^4^^,^^5^

Another complicating factor is that each instrument has different mechanical properties, and a high degree of expertise is required to select the best one for a particular case. Until now, selecting the instrument has been often based on specialist’s experience, which does not always result in a successful procedure.^6^

Recently, the use of computer models to predict the behavior of guidewire and catheter has become increasingly popular.^5^^,^^7^ The purposes of these models include training simulator, preintervention planning (specifically evaluating the performance of an instrument for a specific procedure), and designing instruments.

Despite the growing trend toward computer models, a comprehensive review of different modeling approaches has not yet been performed. Therefore, this article has four goals: (1) to introduce the purposes of guidewire and catheter modeling, (2) to survey different approaches used for instrument modeling and identify their strengths and weaknesses, (3) to study the important factors that affect the interaction between the instrument and the vascular wall, and (4) finally, to review the different strategies used to validate the simulation. We will outline the key areas where future research can improve.

## 2 Review Method

To obtain a comprehensive overview of guidewire and catheter models developed in different studies, we first used Google Scholar as the main search engine and then Web of Science for supplementary information. The keywords were “guidewire,” “catheter,” “modeling,” “simulation,” “training,” “virtual reality,” and “vascular phantom.” Boolean operators (AND, OR, and NOT) were used to combine search terms, and wildcards were applied to deal with spelling variations. Next, criteria for exclusion/inclusion of publications were set, and articles were selected based on their title. Then, the abstract of each selected article was fully read, and the article was either included or excluded based on the relevance and applicability of the content. Finally, to complete the literature search, extra resources from citations and references of the included articles were screened and added when appropriate. In case of duplicate publications, the most recent was included.

## 3 Results

### 3.1 Purposes of Computer Models

**Fig. 1:**
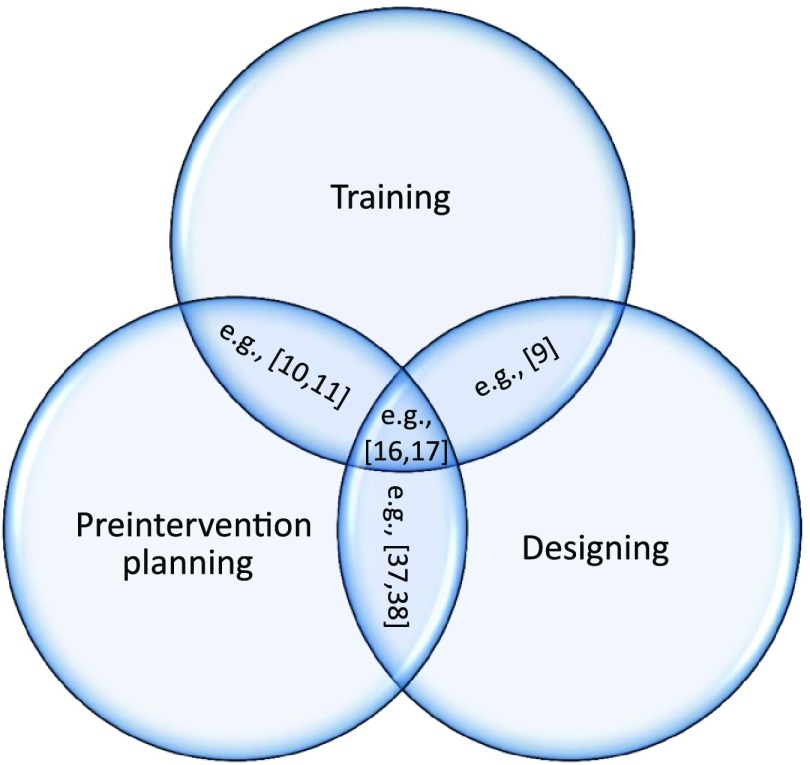
Purposes of a guidewire/catheter model.

In guidewire/catheter modeling, researchers have focused on purposes such as training, preintervention planning, and designing instruments. Although achieving these might overlap (Fig. 1), we will review each one separately.

#### 3.1.1 Training

Simulation-based training is a virtual environment, which helps the specialists to learn complex skills and new catheterization techniques by trial and error without risking patient safety.^7^^,^^8^ In this way, the training becomes more efficient and cost-effective compared to traditional training methods (e.g., using human cadavers and animals). Researchers follow two main approaches: (1) developing a model while focusing on the modeling techniques^9^^–^^32^ and (2) investigating the effectivity and the necessity of using these simulations for training purposes.^7^^,^^8^^,^^33^^–^^36^

#### 3.1.2 Preintervention planning

A simulation can also be used to evaluate the performance of an instrument for a specific anatomy prior to the procedure. This information assists the specialist to select an instrument with the proper mechanical properties and, as a result, increases the success rate of a procedure in accessing the target location. The research done in this field either focuses on catheter^10^^,^^11^^,^^16^^,^^17^^,^^20^^,^^37^^,^^38^ or on guidewire selection.^39^^–^^41^ However, in practice, the instrument selection procedure is still based on the specialist’s experience, which is subjective rather than objective.

#### 3.1.3 Designing instruments

Design optimization of instruments by predicting their behavior inside the body is another purpose of the computer models,^9^^,^^10^^,^^16^^,^^17^^,^^37^^,^^38^^,^^42^^,^^43^ and they are used to test different materials and structures for such instruments and to assess their performance to achieve optimal design. Both numerical (e.g., Ref. 38) and analytical (e.g., Ref. 9) methods have been used to model instrument behavior.

### 3.2 Instrument Modeling

A variety of methods and different techniques have been used to govern the behavior of the instrument in a certain environment.^44^^–^^47^^,^^48^ The following provides an overview of techniques and applied equations and discusses the strengths and weaknesses of each.

#### 3.2.1 Finite-element method

**Fig. 2:**
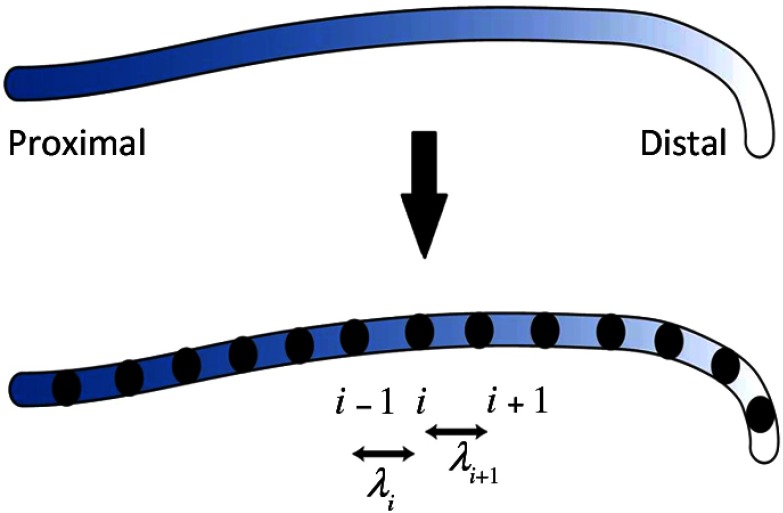
Discretization of the instrument into small segments; $\lambda_{i}$ and $\lambda_{i+1}$ are not necessarily of the same length.

Finite-element method (FEM) is a common numerical technique to model a deformable object,^47^^–^^50^ including the behavior of the guidewire and catheter inside the body.^5^^,^^15^^,^^16^^,^^20^^–^^22^^,^^24^^–^^28^^,^^31^^,^^32^^,^^34^^,^^37^^,^^39^^,^^41^^,^^51^^–^^69^ In this method, the instrument is first divided into a set of basic elements connected by nodes. A function that solves the equilibrium equations is found for each element. The equations incorporate the geometry and material information of the instrument. There are different ways to solve these equations. In Refs. 5, 15, 22, 24–28, 37, 54–66, and 69, the instrument is considered as a rod-like structure, a long and thin circular structure with the length being much larger than the diameter. For rod modeling, there are different choices such as Euler–Bernoulli beam theory (deformation due to bending), Kirchhoff rod,^15^^,^^22^^,^^24^^,^^26^^,^^55^ which is the geometrically nonlinear generalization of the Euler–Bernoulli beam theory,^70^ Timoshenko beam theory (deformation due to bending and shear), and Cosserat rod,^25^^,^^27^^,^^28^^,^^58^^,^^61^^,^^64^^–^^66^^,^^69^ which is the geometrically nonlinear generalization of the Timoshenko beam theory.^70^ In Refs. 15, 22, 37, 41, and 61, the position of the instrument is expressed based on the principles of energy minimization. Thus, the energy function is expressed as

$$E(\mathrm{de})=\min[E_{\mathrm{int}}(\mathrm{de})+E_{\mathrm{ext}}(\mathrm{de})],$$ (1)

where de is the deformation, $E_{\mathrm{int}}$ is the internal energy associated with the flexibility of the instrument, and $E_{\mathrm{ext}}$ is the external energy associated with the applied forces. To solve Eq. (1), the instrument is discretized into multiple segments (see Fig. 2), and the equation is applied to each segment.

FEM is widely used in simulation in different fields because of its numerical stability. Applying this method to model the guidewire and catheter requires a very high computational effort due to the nonlinear underlying effects of FEM.^67^^,^^68^ However, the computational time is highly important and especially in some cases, such as training, being real time is necessary.

#### 3.2.2 Mass-spring model

**Fig. 3:**
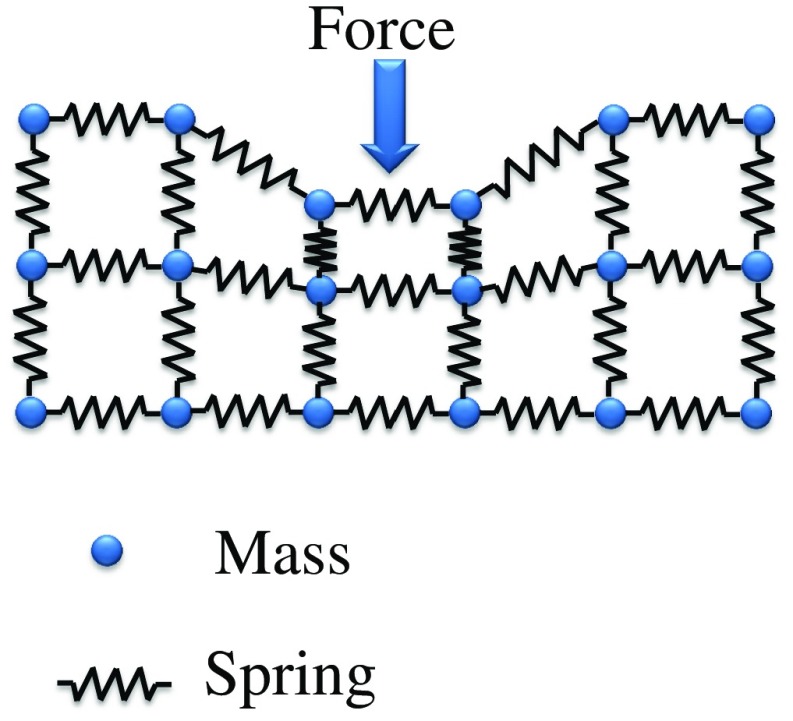
Mass-spring model.

In this method, the instrument is considered as a network of masses connected to each other by springs/dampers (Fig. 3).^12^^,^^23^^,^^44^^,^^45^^,^^71^^–^^74^ The springs not only give flexibility to the model but also constrain the distance between masses. Thus, the number of springs influences the behavior of the model.^74^

The deformable properties of the instrument depend on the parameters of the masses, springs, and dampers as follows:

$$m\overset{¨}{x}=k(x_{0}-x)-d\overset{˙}{x},$$ (2)

where m is the mass of the particle, k is the spring constant related to the stiffness of the instrument, d is the damping coefficient related to the viscous behavior of the instrument, $x_{0}$ is the rest position of the mass, and x is the current position. Thus, concatenating Eq. (2) of all individual masses (N) into a single 3N-dimensional vector and solving them results in the solution for the entire system.

The main advantage of this method is its relative simplicity compared to FEM. However, it is more suited for modeling soft tissue behavior (e.g., the abdominal skin or muscles). In case of a more rigid object, such as the guidewire and catheter, it requires a high computational power, which is against the real-time requirements. Moreover, it is not necessarily accurate, and there is also a risk of numerical instability.^45^^,^^49^

#### 3.2.3 Rigid multibody links

**Fig. 4:**
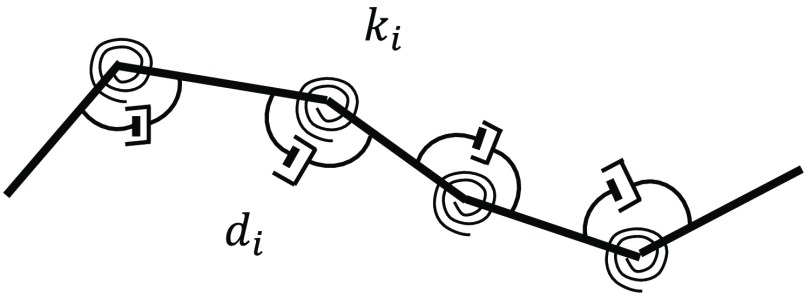
Multiple rigid bodies connected by joints: $k_{i}$ is the spring constant related to the stiffness and $d_{i}$ is the damping coefficient related to the viscous behavior of joint i.

In this method, the instrument is discretized into a set of rigid bodies connected by massless springs and dampers (Fig. 4). The stiffness and damping coefficients are selected based on the material properties of the segments.^75^

In Refs. 13, 18, 29, 38, 40, 76, 77–80, 81, and 82, the instrument is modeled as rigid bodies connected to their neighbors by joints, and the Newton–Euler equations are used to describe the translational and rotational dynamics.^38^^,^^76^^,^^77^^,^^80^^,^^83^ Since the speed of propagating the instrument is slow, the Newton–Euler equations are typically simplified by neglecting inertia and centrifugal force.

In contrast to mass-spring model (MSM), in this method, the length of each segment might be different. Particularly, in guidewire or catheter modeling, it is possible to have shorter segments in the distal side because of more flexibility and longer ones in the proximal side due to more stiffness. This will result in less computational time compared to MSM. Another advantage of this method is that because of its simple structure, it is easy to understand and interpret the results. Moreover, it is relatively easy to incorporate other phenomena such as friction and/or material properties to each individual segment.^43^ On the other hand, the disadvantage of this method is that even though differently sized segment lengths are possible, the simulation is limited to a maximum number of segments, and otherwise it will run into problems.

#### 3.2.4 Hybrid models

The mechanical properties of a guidewire/catheter change along the length, more flexibility at the distal side and more stiffness at the proximal side. Due to this property, some studies came with the idea of applying hybrid models, which means using either a combination of different techniques to model different parts of the instrument^10^^,^^11^^,^^28^^,^^74^^,^^84^^–^^67^ or a new approach that was inspired by different models.^9^^,^^87^^–^^90^^,^^91^^,^^92^^,^^93^ In this way, they endeavored to make the simulation computationally more efficient.

In Ref. 28, the Cosserat rod model is used for the main body and a rigid multibody approach for the flexible tip. Then, the Lagrangian equations of motion are used to solve the dynamics of both parts (body and tip). In Refs. 84 and 86, the flexible tip and the stiff body are modeled by MSM, separately, after which the connection between them is modeled with an additional rigid link (rigid multibody system). In Refs. 10, 11, and 85, the instrument is discretized into a finite number of flexible multibodies. The deformations of bodies are assumed to be relatively small compared to the displacements. Thus, the segments of the instrument are treated as rigid bodies, and displacements are handled by the multibody dynamics approach. Finally, the deformations at their equilibrium position are found by applying FEM.

In Refs. 9, 30, 87–90, 91, 92, and 93, the principles of energy minimization are used to predict the path of the instrument. In contrast to FEM, analytical approximation is applied to solve the optimization problem. In Refs. 9 and 87–90, Hooke’s law^94^ is used as the basis for the modeling. In Refs. 91 and 93, a graph-based modeling is described to find the optimal path for the guidewire in different vascular geometries. Table 1 includes a summary of reviewed models.

**Table 1:** Summary of the reviewed studies.

Modeling technique		Purpose of the model		
		Training	Preintervention planning	Designing
FEM	Kirchhoff rod theory	References 15, 22, 24, 26, and 55		
	Cosserat rod theory	References 25, 27, 58, 61, 65, and 69	Reference 61	Reference 61
	Energy minimization	References 15, 22, 54, and 61	References 41 and 37	Reference 37
	Others	References 16, 21, 32, and 34	References 16 and 39	References 16 and 43
MSM		References 12, 23, 45, and 71–73		
Rigid multibody links		References 13, 18, 19, 29, 77, 79, and 81	References 38, 40, 78, 80, and 82	References 38 and 43
Hybrid		References 9–11, 17, 20, 28, 30, 87–90, 84, 86, and 92	References 10, 11, 17, 20, 85, 91, and 93	References 9–11, 17, 20, and 85

### 3.3 Vessel–Instrument Interaction

The orientation of the instrument is the result of interaction with the vascular wall and is mainly dominated by the forces experienced during propagation. These forces include the manipulation forces, contact forces with the vascular wall, and frictional forces. In this section, our focus is on the contact and frictional forces.

#### 3.3.1 Collision

During the propagation, if the normal distance between the instrument and the vessel is smaller than zero, collision has occurred. Detecting this intersection is referred to as collision detection.

**Fig. 5:**
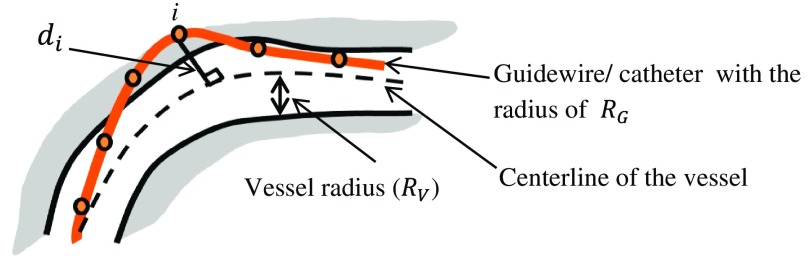
Collision detection.

To detect the collision, some studies^10^^,^^15^^,^^37^^,^^41^^,^^73^^,^^83^^,^^54^ considered a circular cross section for the vessel, in which the radii might vary. Therefore, the shape of the vessel is defined by its centerline and its radius,^95^ and the distance between the instrument and the centerline of the vessel is calculated as follows:

$$D=d_{i}-(R_{v}-R_{G}),$$ (3)

where $d_{i}$ is the nearest point to the centerline of the vessel, and $R_{V}$ and $R_{G}$ are the radii of the vessel and the instrument, respectively (Fig. 5). If D≥0, a contact has been occurred.

In Refs. 10, 16, 17, 37, and 55, the vessel is assumed to be rigid, and no deformation occurs due to the contact. Thus, D is used to calculate the normal force based on Hooke’s law.^94^ In Refs. 83 and 92, vessel deformation is not neglected, and an extra term regarding the reaction force from deformation of the wall in radial direction is considered.

In another collision detection approach, an object is approximated by bounding volumes, and instead of the original object, the intersections of bounding volumes are detected. This method is widely used in simulations.^45^ In Refs. 28, 37, 54, 64, 66, 85, 81, and 92, the axis-aligned bounding boxes method is used, calculating three-dimensional (3-D) boxes that bound the object and using them to test for collision instead of the original object. In Refs. 23 and 55, the object is bounded by spheres instead of boxes. The advantage of this method is less complexity of collision detection and, thus, less computation time. On the other hand, the accuracy depends on the bounding volumes’ size.^45^

#### 3.3.2 Friction

During the propagation of an instrument, friction with the vascular wall influences its orientation^96^ and provides force feedback to the user. For the sake of realism of the simulation, modeling the friction is important. However, the coefficient of the friction is not known from the manufacturers and it is determined empirically. There are two forms of friction: kinetic (or sliding) and static. In Refs. 28, 87–90, 55, 82, and 92, the simulation is based on a quasistatic approach. Therefore, the velocities and accelerations of the instrument in the vessel are small, and the velocity-dependent friction forces are neglected. Although in Refs. 13, 77, 96, and 97 both types of the friction are considered, they did not discuss if a higher accuracy was achieved. Some studies^9^^,^^16^^,^^27^^,^^33^^,^^38^^,^^41^ ignored the friction to trade-off the realism against computation time. However, in reality, friction is not zero, and the instrument that encounters friction results in a different path in the vascular system.^89^^,^^96^

#### 3.3.3 Blood flow

Modeling blood flow can be useful to distinguish between a normal and a narrowed vessel.^34^ However, in most of the studies on guidewire and catheter modeling, the effect of blood flow is neglected to reduce the complexity and only a few studies considered it.^11^^,^^13^^,^^20^^,^^79^^,^^81^ Considering the blood flow when designing catheters with a side hole for the drug delivery might be interesting as the flow condition can affect the injection procedure.^98^ Moreover, in the presence of vascular malformations, modeling the blood flow might provide a better understanding of the pathological conditions.^13^^,^^20^

### 3.4 Validation and Evaluation

The accuracy of any developed model needs to be evaluated. One way to validate a model is by letting a specialist try it out and judge the outcome based on his or her real experiences (face validity).^99^ Most of the reviewed studies validated the simulation results by comparing them with experimental results in phantoms. Phantoms are used both for training and experimental validation. For validation purposes, most of the studies use custom-made phantoms. To fabricate such a phantom, first, they need to extract the vascular geometry in the area of the interest. Thus, Digital Imaging and Communications in Medicine data are obtained via different medical imaging techniques, such as magnetic resonance angiography^15^^,^^100^ and computed tomography.^28^^,^^30^^,^^37^^,^^54^^,^^85^ Then, a variety of segmentation techniques are used to extract the required information. Next, a cast is constructed based on the extracted data. Recently, 3-D-printing has been used to manufacture phantoms.^101^ Different materials can be used to fabricate the phantom model. For example, to test a guidewire or a catheter behavior, phantom’s materials used in the literature include polyvinyl alcohol (PVA),^102^ PVA-hydrogel (PVA-H),^38^^,^^80^^,^^103^ PVA-H and silicone (high transparency),^104^ and PVA-cryogel.^105^

Table 2 includes a summary of some commercially available systems with their applications.

**Table 2:** Example of commercially available systems.

Device/manufacture	Modeling technique	Purpose	Application	Vessel–instrument interaction	Validation method
CathSim, HT Medical Systems^18^^,^^19^^,^^48^	Rigid multibody links	Training	Interventional radiology, and peripheral intravenous	No available information	No available information
da Vinci^16^	FEM	Training, preintervention planning, and designing	Interventional radiology	Rigid vessel wall	Face validity (clinical validation)
				No friction	
ICTS/VIST^13^	Rigid multibody links	Training	Cardiology	Blood flow	No validation based on Ref. 13
				Friction	
ICard^10^^,^^17^	Hybrid^a^	Training, preintervention planning, and designing	Cardiology	Rigid vessel wall	Face validity (clinical validation)
Neuro Cath^11^^,^^20^	Hybrid^a^	Training, preintervention planning, and designing	Neuroradiological procedures	Blood flow	Face validity (clinical validation)
CathI^21^	FEM	Training	Endovascular intervention	Rigid vessel wall	Face validity (clinical validation)

a Refer to Sec. 3.2.

## 4 Discussion and Conclusion

This paper has reviewed the existing computer models for both guidewires and catheters navigation in the body. The purposes of these models are categorized in three main groups: (1) training, (2) preintervention planning, and (3) designing instruments. The main techniques used in the modeling are FEM, MSM, and rigid multibody links. In addition, some studies applied different techniques in different parts of the instrument and introduced hybrid methods. FEM is widely used in simulation in different fields because of its numerical stability. However, due to the nonlinear underlying effects, applying FEM to model the guidewire and catheter requires a very high computational effort. Though MSM is easier than FEM to implement, it is more suited for modeling soft tissue behavior (e.g., the abdominal skin or muscles); modeling a more rigid object, such as the guidewire and catheter, requires high computational power. The rigid multibody links technique has a simple structure that makes it easy to understand and interpret the results and relatively faster than the first two methods. Moreover, adding other phenomena, such as friction and/or material properties, to each individual segment is easy. Therefore, for real-time purposes such as training, the first two methods (FEM and MSM) are not suitable, but for preintervention planning and designing purposes, the FEM and MSM are suitable as well as the rigid multibody links technique.

Capturing all details in one model is a hard task. Thus, each study has made the choice to model only the relevant details for their purpose. One advantage of this selection is that it reduces the computational complexity. However, the results might be biased toward the selected details. Moreover, due to the complexity of the interaction with vessels, different studies have made different assumptions, and therefore, they had to make compromises. For example, in most of the research, the modeling is based on quasistatic mechanics, which is acceptable as the loading of these instruments is slow and inertial effects can be ignored. Some studies assumed a perfect torque control (the torsion coefficient is considered to be infinite). This feature is taken into account in the design of these instruments, and the assumption is close enough to reality. Furthermore, as the manufacturers do not provide information about the mechanical properties of the instruments, this information is determined empirically. Likewise, vessel properties such as diameter, wall thickness, and stiffness, are determined empirically. In many studies, the vessel wall is assumed to be rigid with a circular cross section; thus, deformation of the vessel is not considered. However, a stiff instrument might cause deformation in the vessel. Additionally, the cross section of the vessels might change due to vascular diseases. Therefore, more studies are required to consider different cross sections and to investigate the deformations especially around the tip of the instrument. Validation is the final step in evaluating the accuracy and effectiveness of a model. Face validity, which is done by letting a specialist try it out and judge the outcome based on his or her real experiences, is a reliable method to test a model. However, in practice, most of the reviewed studies have validated their model by comparing the results with phantom experiment results and some of them did not perform any validation. Further, the few reported validation studies that do exist use inconsistent experimental methodologies. Thus, the validation step is a very important one that needs more focus.

The knowledge provided in this review can help to determine a modeling technique for the instrument, which satisfies the necessary requirements for a particular application.
